# Quantification of soluble very low-density lipoprotein receptor in human serum using a sandwich enzyme-linked immunosorbent assay

**DOI:** 10.1016/j.plabm.2023.e00337

**Published:** 2023-09-21

**Authors:** Yoshiharu Tokita, Kazuya Miyashita, Katsuyuki Nakajima, Sadao Takahashi, Akira Tanaka

**Affiliations:** a Faculty of Medical Technology and Clinical Engineering, Gunma University of Health and Welfare, Gunma, Japan; b Kagawa Nutrition University, Tokyo, Japan; c Immuno-biological Laboratories Co, Gunma, Japan; d Department of Clinical Laboratory Medicine, Gunma University, Graduate School of Medicine, Gunma, Japan; e Ageo Central General Hospital, Saitama, Japan; f Takasaki University of Health and Welfare, Gumma, Japan; g Kichijoji Futaba Professional and Vocational College of Culinary Nutrition, Tokyo, Japan

**Keywords:** Soluble very low density lipoprotein receptor (sVLDL-R), Sandwich enzyme-linked immunosorbent assay (ELISA), Body fat percentage, Triglyceride (TG), HbA1c

## Abstract

To investigate the regulation of soluble very low-density lipoprotein receptor (sVLDL-R), which is cleaved mostly from the extracellular domain of VLDL-R II, we generated two rat monoclonal antibodies (mAbs) against human sVLDL-R, and used them to develop a sandwich enzyme-linked immunosorbent assay (ELISA) to measure sVLDL-R levels in human serum or plasma. The ELISA had a linear range from 0.20 ng/mL to 13.02 ng/mL and allowed for the quantification of sVLDL-R in serum and culture cell medium. The coefficient of variation (CV) was less than 10% for both the intra- and inter-assays. The bililubin F, and C, triglyceride (TG), and hemoglobin levels did not affect assay precision. The sVLDL-R concentration was negatively associated with body fat percentage, TG, and HbA1c, suggesting the possibility of obesity and diabetes in middle-aged Japanese women.

## Highlights

• We developed a sandwich ELISA to measure sVLDL-R levels in human serum or plasma.
• The serum sVLDL-R levels was negatively associated with body fat percentage, TG, and HbA1c in middle-aged Japanese women.
• These results suggest that sVLDL-R may be associated with obesity and diabetes.

## 1 Introduction

Very low-density lipoprotein receptor (VLDL-R) is a multifunctional transmembrane protein [1,2]. The soluble ectodomain of VLDL-R (sVLDL-R) is formed by the cleavage of its extracellular domain [1,2]. Takahashi et al. [3] reported the presence of sVLDL-R in human serum and it is known to confer anti-inflammatory and anti-angiogenic effects in ocular tissues by inhibiting canonical Wnt signaling [4]. However, the mechanism through which sVLDL-R is shed into the extracellular space remains unclear. Recently, Ma et al. [5] reported the shedding of sVLDL-R into the extracellular space. They reported novel evidence suggesting that disintegrin and metalloprotease17 (ADAM17) are responsible for sVLDL-R shedding in human pigment epithelial cells by biochemical methods. They also reported that sVLDL-R inhibits fibrosis in neovascular age-related macular degeneration [6].

However, whether sVLDL-R is associated with obesity or diabetes in healthy individuals remains unclear. We generated two rat monoclonal antibodies (mAbs) against human VLDL-R which were then used to develop a sandwich enzyme-linked immunosorbent assay (ELISA) to measure sVLDL-R levels in human serum or plasma. Using the assay, we measured the sVLDL-R concentration in human serum to investigate its association with obesity and diabetes in middle-aged women.

## 2 Materials and methods

### 2.1 Animals

Wister rats (6 weeks old, female, Charles River, MA) were immunized through the subcutaneous and intradermal injection of recombinant human VLDL Receptor/VLDL-R protein (His tag) (ab155621) (Abcam, Tokyo) and complete Freund's adjuvant (GIBCO, MA) at a 1:1 ratio (10μg/0.1 mL). Rats were immunized four times at every other week.

### 2.2 Preparation of hybridoma for the production of soluble VLDL-R antibodies

Two days after the last immunization, lymphocytes from the lymph nodes of rats were used for the preparation of hybridomas. Hybridomas were generated by fusing lymphocytes and myeloma X63-Ag8.653 cells (5:1 ratio). Cell fusion was performed using 50% polyethylenglycol-1500 (Roche, Swiss). Fusion cells were suspended (2.5 × 10^6^ cells/mL as lymphocytes) in HAT medium and seeded onto a 96-well plate (Corning, NY). The plate was incubated at 37 °C for 2 weeks in a 5% CO_2_ incubator and antibody-producing cells were selected. After two weeks, the antibody titer in the supernatant of the culture medium was screened by ELISA. To obtain a high-titer hybridoma, the same procedure was repeated for the selection of the final hybridoma.

### 2.3 Screening

The recombinant human VLDL receptor/VLDL-R protein was diluted to a concentration of 200 μg/mL using phosphate buffered saline (PBS), seeded onto a 96-well ELISA plate (Thermo Fischer Scientifics, MA), and incubated overnight at 4 °C. After washing the plates twice with PBS, blocking solution (200μL/well; PBS with 1% BSA) was added for 1 h. After washing with the blocking solution, the medium was further diluted, seeded onto the ELISA plate and incubated for 30 min at room temperature. After washing the plate four times, peroxidase-labelled anti Rat IgG goat antibody was added to each well (50 μL/well) and incubated for 15 min. After washing five times with washing solution, peroxidase solution (substrate) was added at 50 L/well (Sigma, MO). After 10 min, 1.5 N sulfuric acid was added (50 L/well) and the optical absorbance was measured at 490 nm. As a result, two hybridoma clones that exhibited the highest reactivity to the VLDL receptor/VLDL-R protein, namely14A2 (IgG2a-κ) and 25A3 (IgG1-κ), were selected for ELISA.

### 2.4 Sandwich ELISA

The selected monoclonal antibody 14A2 was diluted with 100 mM bicarbonate buffer (PH 9.5), adjusted to a 5 μg/mL concentration and added to a 96-well microplate at 100μL/well (Nunc, MA). After incubating for two nights at 4 °C, antibody 14A2 was coated onto each well. After washing the plate twice, blocking solution (200μL/well; PBS with 1% BSA) was added and incubated for 1 h. After washing with blocking solution, the calibrator was diluted with the dilution solution (50 μg/mL of 0.5% normal mouse IgG, 1% Triton X-100 mixed with 1% BSA, PBS with 0.05% Tween-20) and was added, along with the samples, to the microplate and incubated overnight at 4 °C. After washing the plate four times with washing solution (10 mM phosphate buffer (pH7.5), 0.05% Tween 20), peroxidase-labelled mAb 25A Fab’ was added to the microplate and incubated for 30 min at 25 °C. After washing five times, the substrate (peroxidase) was added at 100μL/well. After 30 min, 1.5 N sulfuric acid was added and the absorbance was determined at 450 nm.

### 2.5 Study population

The nutrition clinic at Kagawa Nutrition University offered a voluntary 4 month diet and exercise program called the Healthy Diet Course [7] between April 2019 and August 2019. The analyses of the data collected during the program were approved by the Institutional Review Board of Kagawa Nutrition University. Written informed consent was obtained from all of the enrolled participants.

### 2.6 Anthropometric data

Percentage of body fat was measured by dual x-ray absorptiometry (DEXA) (Osteometer, Toyo Medic). Blood samples were collected after overnight fasting at the beginning and end of the program. Samples were allowed to clot for 45 min at room temperature and then centrifuged at 3000 rpm for 10 min at 4 °C. After separation, serum was dispensed into plain microtubes and stored at −80 °C for later analysis. Blood samples were sent to the LSI Medience Corporation for the analyses of the serum levels of liver enzymes, glucose, insulin, HbA1c, lipids and lipoproteins. The pre-heparin serum LPL [8], GPIHBP1 [9] and HTGL [10] concentrations were measured by an ELISA according to the manufacturer's instructions (Immuno-Biological Labs). Briefly, the GPIHBP1 ELISA system was as follows. Without any pretreatment, 10 fold diluted serum samples (100 μl) were incubated with a first antibody (IU-79) coated on the wells at 37 °C for 60 min. Then the GPIHBP1 trapped in the wells was incubated with a secondary antibody (IU-20) at room temperature for 1 h and then reacted with a horseradish peroxidase-labelled antibody against the secondary antibody. Finally GPIHBP1 was detected with substrate solution at 492 nm using a microplate reader. Each sample was measured in duplicate. The intra-assay coefficient of variation was <5%.

### 2.7 Statistical analysis

The data are presented as median values with 25^th^ and 75^th^ percentiles.Accordingly, differences between groups were analyzed using the Mann-Whitney *U* test, and multivariate analyses were performed after logarithmic transformation of the test results for body mass index (BMI), body fat rate, LDL-C, HDL-C, TG, HbA1c, AST, ALT, LPL, and GPIHBP1. Single and multiple regression analyses were performed for each variable, with age as an explanatory variable, to examine the relative contribution of the sVLDLR concentration. All statistical analyses were performed using StatFlex Ver. 6 (Artech, Osaka, Japan). Statistical significance was set at P < 0.05.

## 3 Results

### 3.1 Properties of two new monoclonal antibodies

**Fig. 1:**
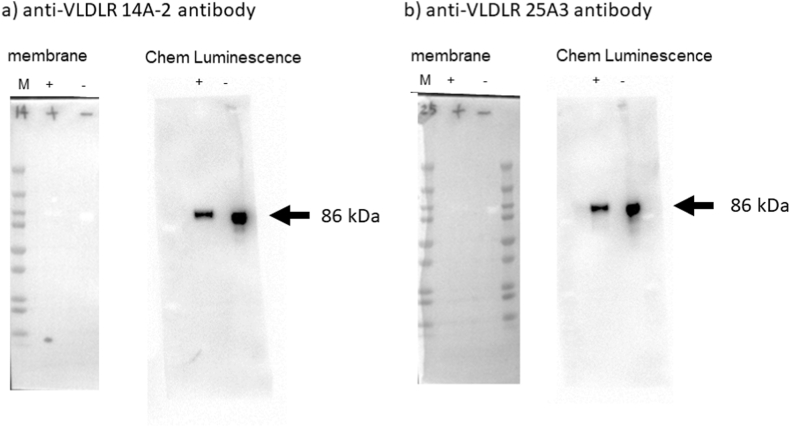
Confirmation of anti-very low-density lipoprotein (VLDL-R) antibody binding to recombinant sVLDLR protein using western blotting. This demonstrates that the anti-VLDLR antibody detected the sVLDLR protein (indicated by arrows in Fig. 1; molecular weight, 86 kDa). The letters in each image are represent the following: M: Molecular size marker, +: with 2 ME, -: without 2 ME.

Fig. 1 shows the results of the western blot analysis for the confirmation of anti-human sVLDL-R. Each sample was pretreated with and without 2-mercaptethernol (2 ME), applied to an acrylamide gel for fractionation, and transferred onto a PVDF membrane. After blocking the membrane, the samples were incubated with anti-sVLDL-R for 120 min at 30 °C in dilution buffer, and further reacted with goat anti-rat-IgG -HRP for 120 min 30 °C and detected sVLDL-R protein using western blot. The monoclonal antibodies 14A2 (IgG2a-κ) and 25A3(IgG1-κ) were used for ELISA. Both monoclonal IgGs bound strongly and specifically to the human sVLDL-R protein with or without 2 ME (Fig. 1).

### 3.2 Development of ELISA for sVLDL receptor

**Fig. 2:**
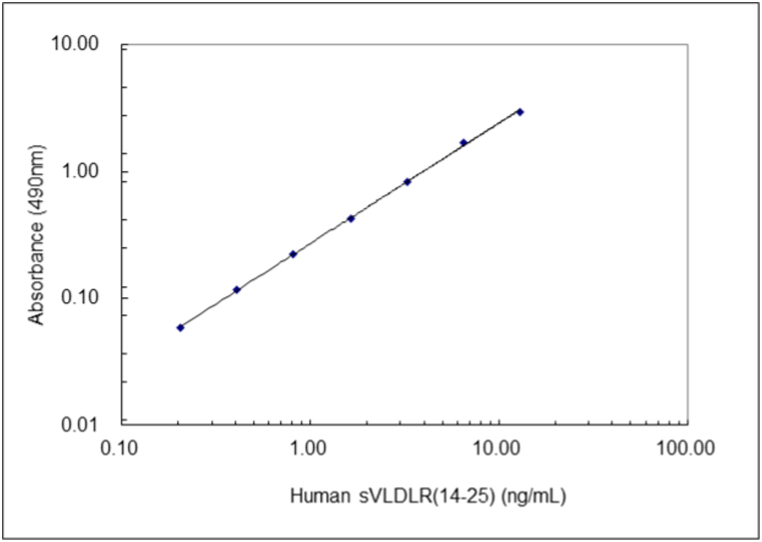
Calibration Curve of Human sVLDLR ELISA.

**Fig. 3:**
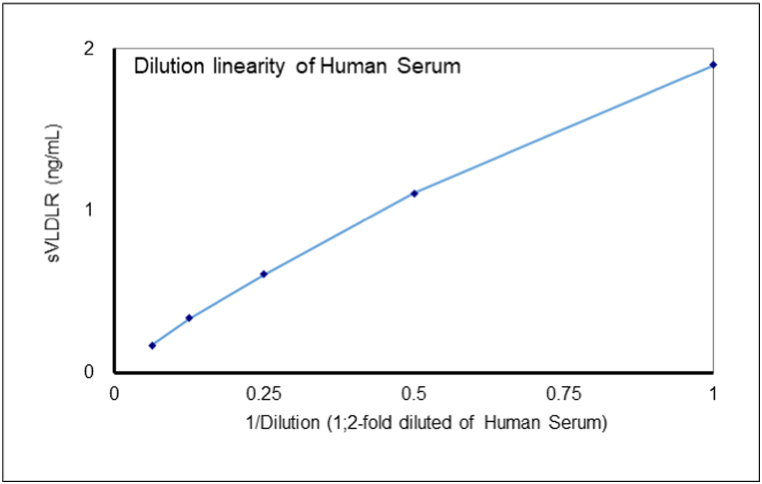
Dilution linearity of sVLDLR ELISA.

We developed a sandwich ELISA for the human sVLDL-R using the 14A2 monoclonal antibody as the capture antibody and the HPR-labelled 25A3 Fab’ monoclonal antibody as the detection antibody. The detactable range of this ELISA was 0.20 ng/mL to 13.02 ng/mL when recombinant human sVLDL-R was used as the calibrator (Fig. 2). The limit of sensitivity for the ELISA was 0.02 ng/mL according to the guidelines of the Clinical & Laboratory Standard Institute. A dilution linearity test indicated that the curve produced linearity by serially diluting human serum parallel to the original standard curve, indicating that this assay system specifically determines the human sVLDL-R concentration (Fig. 3).

Table 1 shows the results of the recovery test, which was performed by adding a human sVLDL receptor standard (0.81 ng/mL to 3.25 ng/mL) to human serum. The recovery rate was between 94% and 99% of the original concentration (Table 1). Interpretations of the inter- and intra-variations of the assay were performed using three quality control samples (high, middle, and low) (Table 2). The coefficients of variation (CVs) of inter-assay imprecision were 4.6, 2.3, and 5.7 in the low, middle and high controls, respectively (Table 2). Moreover, the intra-assay CVs were 2.8, 4.9, and 5.6 in the low, middle and high controls, respectively (Table 2). In addition, interference tests were performed by adding potentially interfering substances, such as bilirubin C and F, triglycerides or hemoglobin, and concentrations up to 500 mg did not affect assay precision.

**Table 1:** Recovery test.

Analyte	Spiked amount (ng/mL)	Expected concentration in spiked sample (ng/mL)	Actual concentration in spiked sample (ng/mL)	Recovery (%)
Human Serum (x5)	3.25	4.20	4.14	98.7
	1.63	2.58	2.47	95.9
	0.81	1.76	1.64	93.5

**Table 2:** Intra- and inter-assay variation.

sVLDL concentration (ng/mL)	Intra-assay variation (n = 4)		Inter-assay variation (n = 3)	
	SD	CV	SD	CV
	(ng/mL)	(%)	(ng/mL)	(%)
6.06	0.29	4.6	0.18	2.8
1.25	0.03	2.3	0.05	4.9
0.39	0.03	5.7	0.03	5.6

**Fig. 4:**
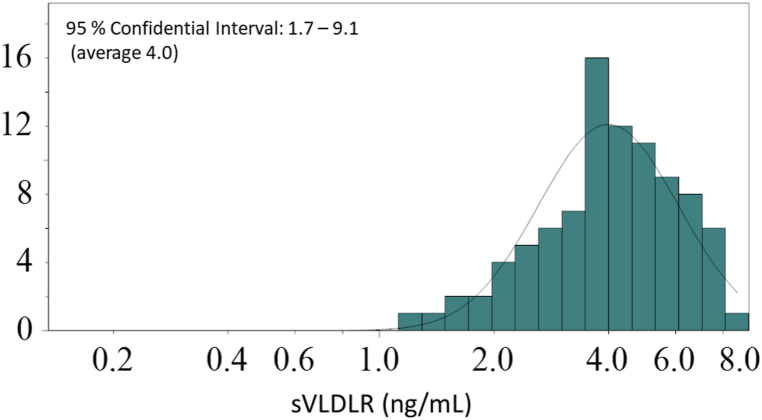
Distribution of sVLDLR concentration in healthy Japanese women (n = 90).

We determined the serum concentrations of sVLDL-R using ELISA and other serum diagnostic markers in healthy female volunteers (Table 3). The reference interval for the sVLDL-R concentration in healthy women was between 1.7 ng/mL and 9.1 ng/mL (n = 90) (Fig. 4).

**Table 3:** Characteristic and serum diagnostic markers in healthy volunteer (n = 90).

	Median	25percentile	75percentile
Age	61	59	67
BMI	27	25.5	30
Abdominal circumference (cm)	94	90	101
Body fat rate (%)	38.2	36.4	43.4
TC (mg/dL)	219	191	238
LDL-C (mg/dL)	127	115	149
HDL-C (mg/dL)	57	48	63
TG (mg/dL)	110	74	154
Insulin resistance	1.5	1.1	2.1
FBS (mg/dL)	88	83	93
HBA1c (NGSP) (%)	5.6	5.5	5.9
SBP (mmHg)	132	123	141
DBP (mmHg)	82	76	88
AST (U/L)	23	19	27
ALT (U/L)	21	16	28
Ch-E (U/L)	395	355	439
ALP (U/L)	223	194	285
gamma-GT (U/L)	27	17	42
Amylase (U/L)	71	57	96
GPIHBP1(pg/mL)	1146	943	1300
sVLDLR (ng/mL)	4.1	3.2	5.5
LPL (ng/mL)	57	45	70

Table 4 shows the results of the single regression analysis of serum diagnostic markers associated with sVLDL-R concentrations in Japanese women. Body fat percentage (P = 0.0109), HbA1c level (p < 0.0001), and TG level (p = 0.0159) were negatively correlated with sVLDL-R. In contrast, the liver markers AST (p = 0.0004) and ALT (p = 0.0123) were positively correlated with sVLDL-R. Multiple regression analysis of age and other parameters, including body fat percentage, TG, HbA1c, AST, and ALT, which were associated with the sVLDL-R concentration in the single regression analysis, were performed (Table 5). These results suggest that sVLDL-R may be associated with obesity and diabetes.

**Table 4:** Single regression analysis of Characteristic and serum diagnostic markers associated with sVLDLR concentration.

	rS	(n = 90)
		P value
Age	0.012	NS
BMI	−0.108	NS
Abdominal circumference (cm)	−0.161	NS
Body fat rate (%)	−0.266	0.0109
TC (mg/dL)	0.156	NS
LDL-C (mg/dL)	0.174	NS
HDL-C (mg/dL)	0.178	NS
TG (mg/dL)	−0.327	0.0159
Insulin resistance	−0.050	NS
FBS (mg/dL)	−0.007	NS
HBA1c (NGSP) (%)	−0.502	<0.0001
SBP (mmHg)	0.063	NS
DBP (mmHg)	0.023	NS
AST (U/L)	0.466	0.0004
ALT (U/L)	0.339	0.0123
Ch-E (U/L)	−0.007	NS
ALP (U/L)	0.034	NS
gamma-GT (U/L)	0.123	NS
Amylase (U/L)	0.099	NS
GPIHBP1(pg/mL)	0.073	NS
LPL (ng/mL)	0.009	NS

**Table 5:** Multiple regression analysis of age and the parameters, including the body fat rate, TG, HbA1c, AST, or ALT, associated with sVLDLR concentration.

Target: sVLDLR	standard beta	t value	(n = 90)
Parameter			p value
Body fat rate (%)	−0.304	3.03992	0.0031
TG (mg/dL)	−0.3015	2.25245	0.0286
HbA1c (NGSP)	−0.5131	3.27748	0.0022
AST (U/L)	0.4845	3.8094	0.0004
ALT (U/L)	0.4227	3.10233	0.0031

## 4 Discussion

VLDL-R has been reported to have two major variants, VLDL-R I and VLDL-R II, which are derived from alternative splicing of *VLDLR* gene. VLDL-R I is the full-length variant, on the other hand, VLDL-R II lacks an O-linked glycosylation domain encoded by exon 16 of *VLDLR* [11,12]. Therefore, sVLDL-R is mainly shed from VLDL-R II. VLDL-R I is mainly expressed in the heart, muscle, and adipose tissue, in contrast, VLDL-R II has dominantly expressed in cerebrum, kidney, spleen, adrenal gland, testis, ovary, and uterus [11]. However, it remains unclear the regulatory mechanism of alternative splicing of *VLDLR* gene in these tissues and whether the these variants have differential physical effects. Magrane et al. have shown that VLDL-R II shedding and releasing its soluble N-terminal extracellular fragment, sVLDL-R, into the culture medium from overexpressed cultured cell rapidly [13], in addition, Takahashi et al. [3] reported the presence of sVLDL-R in human serum. Ma et al. reported that the recombinant sVLDL-R suppresses Wnt signaling [14].

In the present study, we developed a sandwich ELISA to measure human serum sVLDL-R concentrations. sVLDL-R is the extracellular domain cleaved from VLDL-R and is soluble in serum. The ELISA used two monoclonal antibodies against human sVLDL-R. ELISA accurately determined the concentration of sVLDL-R, which is shed by the cleavage of the extracellular domain of VLDL-R [[1], [2], [3]]. This assay is sensitive using specific monoclonal antibodies and can be used to measure normal human serum sVLDL-R levels. We investigated the association between sVLDL-R levels and other clinical parameters in Japanese individuals with and without obesity who underwent dietary and exercise interventions [15]. Single regression analysis revealed an association between sVLDL-R and body fat percentage as well as TG, HbA1c, ALT, and AST levels. sVLDL-R was negatively associated with body fat percentage and TG and HbA1c levels, indicating the possibility of diabetes, whereas ALT and AST were positively associated. These correlations were confirmed using age-adjusted multiple regression analysis.

Further studies are required to elucidate the relationship between sVLDL-R levels and diabetes. We plan to investigate the relationship among sVLDL-R, GPI-HBP1, and LPL.

In conclusion, we developed a specific and sensitive sandwich ELISA to measure sVLDL-R concentrations in human serum. This newly developed assay will prove an useful tool for in vivo and in vitro research efforts to elucidate the mechanisms underlying the regulation of the VLDL receptor and its role in sVLDL-R.

## Author contribution statement

**YT**: Formal analysis, Investigation, Writing, Project administration, and Funding acquisition **KM**: Methodology, Validation, and Resources **KN**: Conceptualization and Writing **ST**: Conceptualization and Supervision **AT**: Conceptualization, Supervision, and Funding acquisition.

## Declaration of competing interest

The authors declare that they have no known competing financial interests or personal relationships that could have appeared to influence the work reported in this paper.

## Data Availability

Data will be made available on request.
